# A predictive model of serum calcium and 25-hydroxyvitamin D for recurrence of benign paroxysmal positional vertigo in the elderly: a single-center follow-up cohort study

**DOI:** 10.3389/fneur.2026.1865876

**Published:** 2026-09-18

**Authors:** Xue Zhang, Xizuo Pu, Yingmei Wang, Xingya Li, Qingchun Pan

**Affiliations:** 1 Affiliated Hospital of North Sichuan Medical College, Nanchong, Sichuan, China; 2 Longchang People's Hospital, Longchang, Sichuan, China

**Keywords:** 25-hydroxyvitamin D, benign paroxysmal positional vertigo, canalith repositioning procedure, prediction model, recurrence, serum calcium

## Abstract

**Objectives:**

To investigate the predictive value of serum calcium and 25-hydroxyvitamin D (25-OH-VD) on recurrence after canalith repositioning procedure (CRP) in elderly patients with benign paroxysmal positional vertigo (BPPV), and to establish a quantitative prediction model.

**Methods:**

A single-center follow-up cohort study was conducted involving 286 elderly BPPV patients (age≥60 years) treated with CRP from January 2020 to June 2025. Patients were followed for 1 year and divided into a non-recurrence group (*n* = 213) and a recurrence group (*n* = 73) based on recurrence status. Additionally, 60 healthy elderly individuals served as controls for comparing baseline biochemical levels. Fasting serum 25-OH-VD and calcium levels were measured. Pearson correlation analysis evaluated the relationship between variables. Receiver operating characteristic (ROC) curves assessed predictive performance, and multivariate logistic regression identified independent risk factors to establish a predictive model.

**Results:**

Serum calcium (1.94 ± 0.47 mmol/L) and 25-OH-VD (15.34 ± 3.45 ng/mL) levels were significantly lower in the recurrence group compared to the non-recurrence group (2.35 ± 0.56 mmol/L, 18.44 ± 3.74 ng/mL) and controls (2.68 ± 0.48 mmol/L, 23.61 ± 3.92 ng/mL; all *p <* 0.05). Levels were positively correlated (*r* = 0.637, *p* < 0.05). Multivariate logistic regression revealed that reduced serum calcium (*OR* = 0.301, 95%*CI*: 0.152–0.593), reduced 25-OH-VD (*OR* = 0.808, 95%CI: 0.728–0.898), multiple repositioning maneuvers (*OR* = 4.945), hypertension (*OR* = 2.237), sleep disorders (*OR* = 2.113), cervical spondylosis (*OR* = 2.167), and anxiety (OR = 2.726) were independent risk factors for recurrence. The combined predictive model yielded an AUC of 0.762 (95%*CI*: 0.697–0.827), superior to single indicators (calcium AUC = 0.707; 25-OH-VD AUC = 0.717). The optimal cut-off value was 0.339 (sensitivity 56.2%, specificity 81.2%).

**Conclusion:**

Serum calcium and reduced 25-OH-VD are independent predictive factors for post-CRP recurrence in elderly BPPV patients, and their combination improves predictive efficacy. The predictive model provides a quantitative basis for early identification of high-risk patients. However, due to its relatively low sensitivity, the model should be used as an adjunctive tool rather than a standalone test in clinical practice. These findings require further external validation in multi-center studies, and interventional effects of calcium and vitamin D supplementation should be confirmed by randomized controlled trials.

## 1 Introduction

Benign Paroxysmal Positional Vertigo (BPPV) is a highly prevalent peripheral vestibular dysfunction in clinical practice. Its typical feature is transient rotational vertigo and characteristic nystagmus that is triggered by specific head position changes relative to gravity, appears after a brief latency, and fatigues within 30–60 s (1). Global epidemiological data show that the BPPV’s lifetime prevalence is about 2.4%, and the incidence raises significantly with age; the incidence in the elderly population can be more than three times that of the younger population, and it has become the primary cause of vertigo in the elderly (2). BPPV not only provokes severe vertiginous episodes but also markedly elevates fall risk secondary to impaired balance, which may result in serious complications including fractures and craniocerebral trauma, thereby imposing a substantial burden on elderly patients’ quality of life, independent mobility and the public health system; moreover, emerging evidence demonstrates a significant association between BPPV and comorbid psychiatric symptoms—particularly anxiety and depression—which further compromise daily functioning and health-related quality of life (3).

Beyond its mechanical etiology, emerging evidence highlights the importance of vitamin D in maintaining inner ear homeostasis and overall vestibular health, providing a conceptual framework for understanding metabolic influences on BPPV recurrence (4, 5).

At present, canalith repositioning procedure (CRP) is included in the list of first-line interventions in BPPV, by a sequence of variations in head position to cause the detached otoconia to re-enter the utricle, resulting in mechanical repositioning, and the short-term success rate may be up to 70% ~ 90% (6). Nonetheless, clinical experience has discovered that despite the successful resolution of positional nystagmus by CRP, 31% ~ 61% of patients continue to experience the risk of disease recurrence, and the elderly population, with multiple comorbidities and diminished vestibular functioning, has a higher recurrence rate and more adverse outcomes (7). Recurrence is usually characterized by the reoccurrence or continuation of vertigo symptoms, and its pathogenesis is multifactorial, including delayed vestibular compensation, abnormal central integration, and imbalance of multisensory input (8). It is also worth mentioning that recurrence does not only extend the symptom duration of patients but can also, by restricting activities and causing fear of falling, worsen functional decline, creating a vicious cycle. Thus, in elderly BPPV patients, the predictive factors of recurrence and early intervention are of critical clinical importance to enhance the prognosis, particularly, the necessity to investigate quantifiable and readily available biomarkers.

The pathology of BPPV is directly connected to the otolith metabolic disorders. The gelatinous membrane contains otoliths, which are calcium carbonate crystals in the inner ear, and their stability is extremely reliant on the local calcium ion homeostasis (9). Vitamin D is a central regulatory element in calcium and phosphorus metabolism; it controls the expression of epithelial calcium channels (including ECaC1) directly influencing the endolymph calcium level and the process of otolith crystal formation and dissolution (10). Research has established that a lack of vitamin D is associated with abnormal calcium metabolism in the inner ear, which may contribute to otolith dissolution or abnormal detachment, thereby increasing the risk of BPPV (11). Clinical observational studies also suggest that lower serum 25-hydroxyvitamin D (25-OH-VD) levels are strongly linked to the incidence and the risk of recurrence of BPPV, and vitamin D supplementation is very effective in lowering the recurrence rate (12). Simultaneously, serum calcium, being a direct measure of systemic calcium metabolism, its deviation can indicate a decrease in the stability of otolith minerals (13). Vitamin D insufficiency or deficiency is prevalent among older adults due to age-related changes, reduced sunlight exposure and inadequate nutritional intake (with over half of elderly individuals presenting 25-OH-VD levels < 20 ng/mL), while osteoporosis and osteopenia are also common; these metabolic bone disorders are of particular relevance to geriatric BPPV because they reflect systemic calcium and bone metabolic disturbances that may directly compromise otolith integrity, and existing evidence has demonstrated that osteoporosis is associated with a higher incidence of BPPV and low bone mineral density represents a potential risk factor for both BPPV onset and recurrence, collectively supporting the contribution of metabolic bone disease to vestibular disorders (14). These metabolic abnormalities can potentially act synergistically to facilitate the occurrence and progression of BPPV, but their contribution to recurrence has not been systematically clarified, particularly without specific assessment in the elderly population, and serum calcium and 25-OH-VD as readily measurable biochemical markers may offer new methods of predicting recurrence.

Despite the clinical importance of BPPV recurrence, simple, objective biomarkers to predict its occurrence are currently lacking. The current literature is largely concerned with the clinical presentation of recurrence or parameters of vestibular functions, but it does not pay attention to the predictive power of biochemical markers (15, 16). The elderly patients are frequently comorbid (hypertension, diabetes, hyperlipidemia, etc.), and these conditions can worsen the risk of recurrence by influencing the microcirculation or metabolic conditions of the inner ear (17). Nevertheless, the link between 25-OH-VD and serum calcium and recurrence in elderly BPPV patients has not been systematically assessed, and no threshold analysis of their predictive ability has been published. The elucidation of the correlation between these indicators and recurrence not only contributes to the disclosure of the pathophysiological mechanism of recurrence, but also offers clinically measurable risk assessment tools, which will guide individualized prevention and treatment plans (including specific supplementation of calcium or vitamin D), which will reduce the number of falls and improve the long-term prognosis of patients.

Based on this, this study, through a single-center follow-up cohort study design, analyzes systematically the correlation of serum calcium and 25-OH-VD in elderly BPPV patients with post-CRP recurrence, and uses the ROC curve to measure its predictive performance and optimal cutoff value. The research findings are expected to provide new ideas for early identification and intervention of recurrence, and to improve the overall management level of elderly BPPV.

## 2 Materials and methods

### 2.1 Research subjects

This study was designed as a single-center follow-up cohort study. From January 2020 to June 2025, elderly BPPV patients who received CRP at our hospital were enrolled. Initially, 320 patients were assessed for eligibility, 34 were excluded due to failure of CRP or other reasons, and 286 completed the 1-year follow-up with no loss to follow-up. Inclusion criteria (16): (1) patients who met the BPPV diagnosis and treatment guidelines (2017), and were over 60 years old and able to cooperate with various examinations; (2) signed informed consent form; (3) vertigo induced by changes in head position; (4) Characteristic positional nystagmus corresponding to the affected semicircular canal, specifically: BPPV involving the posterior, horizontal (lateral), or anterior semicircular canals was included in this study. (a) Posterior semicircular canal: vertical-torsional nystagmus on Dix-Hallpike test; (b)Horizontal semicircular canal: horizontal geotropic or apogeotropic nystagmus on supine roll test; (c)Anterior semicircular canal: downbeat nystagmus on Dix-Hallpike test; (5) no other CNS diseases; (6) No other vestibular dysfunction, no thyroid diseases, no gynecological diseases, and no use of hormonal drugs within 3 months prior to enrollment; (7) treated according to the BPPV diagnosis and treatment protocol, with positional nystagmus disappearing after treatment. (8) No use of calcium, vitamin D, or osteoporosis drugs after efficacious CRP treatment.

**The exclusion criteria are:** (1) history of craniocerebral injury; (2) secondary BPPV patients, including ear diseases, vestibular neuritis, vestibular migraine, Ménière’s disease, etc.; (3) central nervous system damage; (4) other factors impacting brain function and structure; (5) comorbid psychiatric or neurological diseases, and patients unable to cooperate with work. (6) those who had received vitamin D, calcium treatment, or osteoporosis treatment. (7) severe hepatic or renal dysfunction; (8) parathyroid disorders; (9) osteoporosis; (10) use of medications affecting calcium or vitamin D metabolism (e.g., thiazide diuretics, anticonvulsants, bisphosphonates, vitamin D/calcium supplements within the past 3 months).

The control group was recruited from the physical examination center of our hospital during the same period. Controls were matched for age and sex with the BPPV patients. Inclusion criteria for controls were: (1) no history of BPPV or other vestibular disorders; (2) no gynecological or thyroid diseases; (3) no use of hormonal drugs, calcium, or vitamin D supplements within 3 months prior to enrollment; (4) normal results on Dix-Hallpike and supine roll tests.

In addition to positional tests, all patients underwent comprehensive vestibular function assessments, including caloric testing and video head impulse test (vHIT) using VNG goggles, to rule out other peripheral vestibular disorders. However, these vestibular test results were not included in the recurrence risk analyses.

This study was approved by the Ethics Committee of the Affiliated Hospital of North Sichuan Medical College (Approval No. 2020ER723-1; approved on January 15, 2020). All participants provided written informed consent, and all participants were fully informed about the examination and treatment contents and consented (Figure 1).

**Figure 1:**
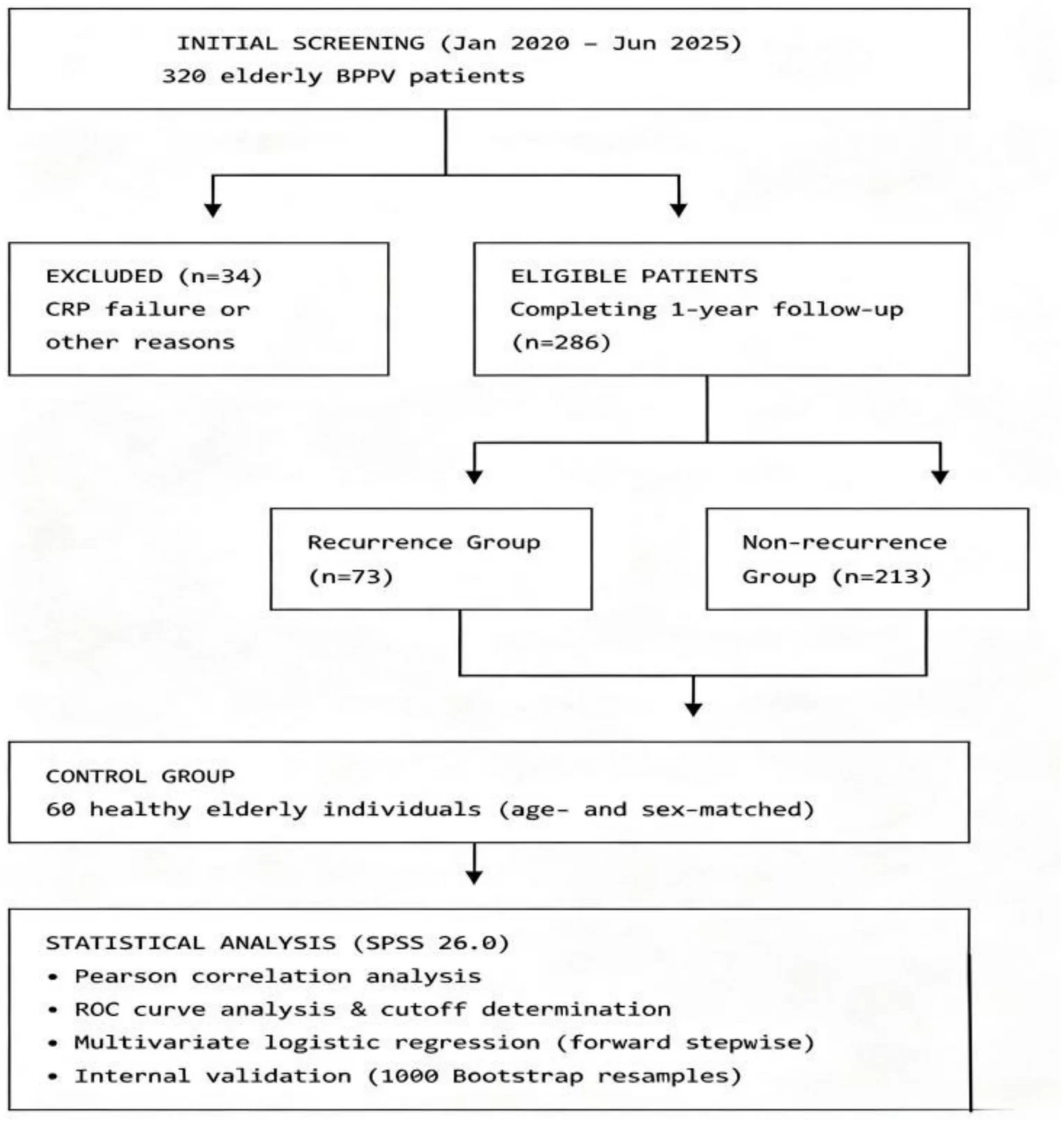
Participant screening and study flow diagram.

### 2.2 BPPV diagnosis and treatment

The diagnosis of BPPV is based on the Bárány Society diagnostic criteria (18): The diagnostic criteria included: (1) recurrent episodes of vertigo triggered by changes in head position relative to gravity; (2) duration of vertigo episodes<1 min; (3) characteristic positional nystagmus observed during positional testing; (4) absence of other neurological or otological signs indicating central or other peripheral vestibular pathology. For the posterior semicircular canal, vertical-torsional nystagmus was observed during the Dix-Hallpike test; for the horizontal semicircular canal, horizontal geotropic or apogeotropic nystagmus was observed during the supine roll test; for the anterior semicircular canal, downbeat nystagmus was observed during the Dix-Hallpike test. CRP treatment for BPPV was performed as follows (19): (1) Epley method for posterior semicircular canal patients; (2) Yacovino method for anterior semicircular canal patients; (3) Combined treatment of Epley method and Barbecue method for multi-canal BPPV.

While positional tests (Dix-Hallpike and supine roll tests) were essential for BPPV diagnosis, additional vestibular function test results were not included in the prediction model. This decision was primarily driven by clinical practicality: serum calcium and 25-OH-VD are readily available, cost-effective biochemical markers that can be easily measured during routine follow-up. For primary care physicians and general practitioners who may not have access to specialized vestibular testing equipment, these biomarkers provide a more accessible tool for identifying patients at high risk of recurrence.

### 2.3 Efficacy evaluation

CRP efficacy evaluation (20): Based on the 2017 published BPPV treatment and diagnosis criteria, according to the positional nystagmus and vertigo responses exhibited by patients, the following classifications can be determined: (1) Cured: vertigo symptoms completely absent; (2) Improved: vertigo and nystagmus are relieved, but not completely eliminated; (3) Ineffective: no sign of relief of vertigo or nystagmus, and even deterioration occurs.

Evaluation of recurrence: (1) Regular follow-up: a systematic evaluation is conducted every 3 months; (2) Follow-up method: combining outpatient re-examination (main) and structured telephone follow-up (auxiliary); (3) The definition of recurrence must meet all of the following conditions: symptoms reappear within 12 months after CRP treatment, typical clinical manifestations conforming to the 2017 BPPV diagnosis and treatment guidelines, confirmed diagnosis records from this hospital and other medical institutions, positional test inducing characteristic nystagmus.

### 2.4 Serum calcium and 25-OH-VD test

All subjects had 5 mL of venous blood collected from their elbows in the morning on an empty stomach (8–12 h of fasting). The control group had blood collected on the day of enrollment, while the experimental group had blood collected within 24 h of BPPV diagnosis. Vacuum blood collection tubes without anticoagulants (containing coagulants) were used to collect samples. The samples were strictly centrifuged (centrifugation radius 15 cm; 3,000 r/min; 15 min) within 2 h after collection. The supernatant was separated, aliquoted into EP tubes, and stored in an ultra-lower temperature freezer at −80 °C for later testing. The detection method adopted internationally recognized standard technology: The 25-OH-VD concentration was determined using the Roche Cobas e601 ECLIA method (Roche Diagnostics GmbH, Mannheim, Germany). Serum calcium was detected using a Hitachi 7,600 fully automated biochemical analyzer (Hitachi High-Tech Corporation, Tokyo, Japan; azoarsium III method). The classification was formulated with reference to authoritative guidelines at home and abroad (21): 25-OH-VD was divided into four levels: severe deficiency (<12 ng/mL), sufficient (20–100 ng/mL), insufficient (12–20 ng/mL), and risk of poisoning (>100 ng/mL). <20 ng/mL was defined as an abnormal vitamin D state. Serum calcium was detected using a Hitachi 7,600 fully automated biochemical analyzer (azoarsium III method). High and low concentration quality control samples were set for each batch of tests to ensure accuracy. Serum calcium concentration was determined with reference to the normal range of the laboratory (2.10–2.55 mmol/L) (22).

### 2.5 Related indicators

All comorbid conditions were diagnosed and confirmed by experienced specialists based on established diagnostic criteria, medical history, physical examination, and relevant auxiliary tests.

The selection of comorbidities in this study was based on their previously reported associations with BPPV recurrence. Clinical evidence suggests that metabolic disorders (hypertension, hyperlipidemia, hyperglycemia) may impair inner ear microcirculation or alter endolymph pH, thereby affecting otoconia stability. Additionally, sleep disorders, cervical spondylosis, migraine, anxiety, and depression may influence vestibular compensation or increase recurrence risk through autonomic, mechanical, or psychological pathways (23, 24). To ensure standardized assessment, the following diagnostic criteria were applied:

Hypertension (25): Hypertension is described as SBP ≥ 140 mmHg and/or DBP ≥ 90 mmHg, or being treated with antihypertensive medications. Hyperlipidemia (26): Hyperlipidemia includes TC ≥ 5.2 mmol/L, TG ≥ 1.7 mmol/L, LDL-C ≥ 3.4 mmol/L, or HDL-C < 1.0 mmol/L (male) or <1.3 mmol/L (female). Hyperglycemia (27): Hyperglycemia is defined as ≥7.0 mmol/L fasting blood glucose, 2-h ≥ 11.1 mmol/L post-glucose load blood glucose, or ≥6.5% glycated hemoglobin (HbA1c). Sleep disorders (28): Diagnostic criteria meet the definition of the International Classification of Sleep Disorders (ICSD-3): difficulty falling asleep (sleep onset time > 30 min); ≥ 2 nighttime awakenings or early awakening; total sleep time < 6 h; accompanied by daytime dysfunction (such as fatigue, decreased attention) lasting ≥ 1 month.

Cervical spondylosis (29): Cervical spondylosis is degenerative variations in the cervical spine (i.e., intervertebral disc herniation and osteophyte formation) shown on imaging (X-ray/CT/MRI), accompanied by symptoms such as radiating pain in the upper limbs, limited neck movement, or instability when holding objects.

Migraine (30): Meets the International Headache Society (IHS) criteria: recurrent throbbing headache (4 to 72 h); accompanied by the following two features: unilateral headache, throbbing pain, moderate to severe pain (affecting daily activities), nausea/vomiting or photophobia/phonophobia; no other evidence of intracranial organic disease.

Anxiety (31): Diagnostic criteria: in accordance with the Diagnostic and Statistical Manual of Mental Disorders (DSM-5): Generalized anxiety disorder (GAD): excessive worry lasting more than 6 months, accompanied by muscle tension, irritability, etc.; panic disorder: sudden onset of intense fear accompanied by a sense of impending death, ≥1 episode per month.

Depression (32): Diagnostic criteria: in accordance with the Diagnostic and Statistical Manual of Mental Disorders (DSM-5): Major depressive disorder (MDD): persistent low mood and loss of interest for more than 2 weeks, accompanied by ≥4 somatic symptoms (such as sleep disturbances, fatigue); Depressive state: significantly depressive mood affecting function, but not meeting the diagnostic criteria for MDD.

### 2.6 Statistical methods

Data were analyzed using SPSS 26.0 software (IBM Corp., Armonk, NY, USA). The Shapiro–Wilk test was used to assess normality. Measurement data conforming to normal distribution are expressed as mean ± SD and compared using independent t-tests (two groups) or one-way ANOVA (multiple groups). Non-normally distributed data are presented as median (interquartile range) and compared using the Mann–Whitney U test or Kruskal–Wallis test. Pearson correlation analysis was used to evaluate the relationship between serum calcium and 25-OH-VD levels. Univariate and multivariate logistic regression analyses were performed to identify risk factors for recurrence. Variables with *p* < 0.05 in univariate analysis were entered into a multivariate model using forward stepwise selection (likelihood ratio). Collinearity was assessed using variance inflation factor (VIF), with VIF > 5 indicating significant multicollinearity. ROC curve analysis was used to evaluate the predictive value of serum calcium and 25-OH-VD for recurrence, and the optimal cutoff values were determined by maximizing the Youden index. The 60 healthy controls were used only for comparing baseline biochemical levels with BPPV patients and were not included in the recurrence prediction model, which was developed exclusively from the 286 BPPV patients. The final prediction model included only serum calcium and 25-OH-VD. Internal validation was performed using Bootstrap resampling (1,000 iterations) to assess overfitting and optimism correction. Model performance was evaluated by AUC, calibration curve (calibration intercept and slope), Brier score, and decision curve analysis (DCA). No missing data were present for any variables in the model. Two-sided *p* < 0.05 was considered statistically significant.

## 3 Result

### 3.1 Comparison of general data between the experimental group and the control group

This study enrolled 286 elderly BPPV patients who completed 1-year follow-up, with a median age of 65 (63, 69) years. Posterior semicircular canal involvement was identified in 205 patients (71.7%), and horizontal semicircular canal involvement in 81 patients (28.3%); no isolated anterior canal BPPV was recorded. Seventy-three patients developed post-CRP recurrence, and 213 remained recurrence-free, corresponding to a recurrence rate of 25.52%. The control group consisted of 60 elderly subjects aged 64.5 (62, 68) years.

As summarized in Table 1, baseline characteristics were balanced among the three groups, with no significant differences in age or sex (all *p* > 0.05). Both BPPV subgroups exhibited significantly higher prevalence of hypertension, hyperlipidemia and hyperglycemia than the control group (*p* < 0.05). Additionally, patients in the recurrence group were more likely to require multiple repositioning maneuvers than those without recurrence (*p* < 0.05). Univariate analysis demonstrated significant differences between recurrent and non-recurrent BPPV patients in the number of repositioning attempts, hypertension, hyperlipidemia, hyperglycemia, sleep disorders, cervical spondylosis, anxiety, as well as serum calcium and 25-OH-VD levels (Table 1).

**Table 1:** Comparison of baseline characteristics among control, non-recurrence, and recurrence groups.

Variables	Control group (*n* = 60)	Experimental group (*n* = 286)		*χ^2^/t/z*	*p*
		No recurrence group (*n* = 213)	Relapse group (*n* = 73)		
Age (years)	64 (63, 66)	64 (62, 66)	65 (63, 66)	−0.636	0.525
Sex (Male/Female, *n* %)				0.631	0.427
Male	26 (43.33)	96 (45.07)	29 (39.73)		
Female	34 (56.67)	117 (54.93)	44 (60.27)		
Affected semicircular canals	—			0.159	0.690
Horizontal semicircular canal	—	59 (27.7)	22 (30.14)		
Posterior semicircular canal	—	154 (72.3)	51 (69.86)		
Anterior semicircular canal	—	0	0		
Number of repositioning maneuvers				23.012	<0.001 ***
1 Second-rate	—	149 (69.95)	28 (38.36)		
Repeatedly	—	64 (30.05)	45 (61.64)		
Hypertension (%)				9.998	0.002 **
None	42 (70.00)	130 (61.03)	29 (39.73)		
Have	18 (30.00)	83 (38.97)	44 (60.27)		
High blood lipids (%)				4.403	0.036 *
None	41 (68.33)	132 (61.97)	35 (47.95)		
Have	19 (31.67)	81 (38.03)	38 (52.05)		
High blood sugar (%)				5.566	0.018 *
None	46 (76.67)	141 (66.20)	37 (50.68)		
Have	14 (23.33)	72 (33.80)	36 (49.32)		
Sleep disorders (%)				15.274	<0.001 ***
None	37 (61.67)	132 (61.97)	26 (35.62)		
Have	23 (38.33)	81 (38.03)	47 (64.38)		
Cervical spondylosis (%)				10.137	0.001 ***
None	44 (73.33)	149 (69.95)	36 (49.32)		
Have	16 (26.67)	64 (30.05)	37 (50.68)		
Migraine (%)				0.490	0.484
None	52 (86.67)	185 (86.85)	61 (83.56)		
Have	8 (13.33)	28 (13.15)	12 (16.44)		
Anxiety (%)				17.854	<0.001 ***
None	53 (88.33)	181 (84.98)	45 (61.64)		
Have	7 (11.67)	32 (15.02)	28 (38.36)		
Depression (%)				1.128	0.288
None	56 (93.33)	198 (92.96)	65 (89.04)		
Have	4 (6.67)	15 (7.04)	8 (10.96)		
Serum calcium (mmol/L)	2.68 ± 0.48	2.35 ± 0.56	1.94 ± 0.47	5.580	<0.001 ***
25-OH-VD (ng/ml)	23.61 ± 3.92	18.44 ± 3.74	15.34 ± 3.45	6.238	<0.001 ***

BPPV, benign paroxysmal positional vertigo; CRP, canalith repositioning procedure; 25-OH-VD, 25-hydroxyvitamin D; SD, standard deviation; M, Median. The healthy control group was used only for baseline comparisons of serum calcium and 25-OH-VD levels; recurrence risk factor analysis was performed exclusively in BPPV patients (non-recurrence vs. recurrence). Data are shown as mean ± SD or *n* (%). Normally distributed continuous variables were compared using one-way ANOVA with LSD post-hoc test, and categorical variables were analyzed using the chi-square test. *p < 0.05, **p < 0.01, ***p < 0.001; #*p* < 0.05 vs control group; #*p* < 0.05 vs non-recurrence group.

MLR analysis regression analysis was conducted using the number of repositioning attempts, hypertension, hyperglycemia, hyperlipidemia, sleep disorders, cervical spondylosis, anxiety, serum calcium, and 25-OH-VD as independent variables, and BPPV recurrence as the dependent variable. The number of repositioning manoeuvres was defined as the total number of CRP sessions required to achieve complete resolution of positional nystagmus: a single manoeuvre (1 session) versus multiple manoeuvres (≥2 sessions performed on separate visits).

In the multivariate logistic regression analysis (Table 2), multiple repositioning manoeuvres (*OR* = 4.945, 95% *CI*: 2.472–9.891, *p* < 0.001), hypertension (*OR* = 2.237, 95% *CI*: 1.127–4.442, *p* = 0.021), sleep disorders (*OR* = 2.113, 95% CI: 1.073–4.163, *p* = 0.031), cervical spondylosis (*OR* = 2.167, 95% *CI*: 1.078–4.359, *p* = 0.003), and anxiety (*OR* = 2.726, 95% *CI*: 1.264–5.880, *p* = 0.011) were identified as independent risk factors for recurrence. Conversely, reduced serum calcium (*OR* = 0.301, 95% *CI*: 0.152–0.593, *p* = 0.001) and reduced 25-OH-VD (*OR* = 0.808, 95% *CI:* 0.728–0.898, *p* < 0.001) were significantly associated with lower odds of recurrence per unit increase, indicating that lower levels of these biomarkers confer a higher risk of BPPV recurrence. All VIF values were < 5, indicating no significant multicollinearity.

**Table 2:** Logistic regression analysis of risk factors for recurrence after CRP in the experimental group.

Variables	*B*	*S. E.*	Wald	*P*	*OR*	95% *CI*		Collinearity diagnostics	
						Lower limit	Upper limit	Tolerance	*VIF*
Number of repositioning maneuvers	1.598	0.354	20.418	<0.001	4.945	2.472	9.891	0.955	1.047
Hypertension	0.805	0.350	5.297	0.021	2.237	1.127	4.442	0.940	1.064
Sleep disorders	0.748	0.346	4.680	0.031	2.113	1.073	4.163	0.937	1.068
Cervical spondylosis	0.774	0.356	4.710	0.003	2.167	1.078	4.359	0.964	1.037
Anxiety	1.003	0.392	6.540	0.011	2.726	1.264	5.880	0.933	1.072
Serum calcium	−1.202	0.346	12.05	0.001	0.301	0.152	0.593	0.901	1.110
25-OH-VD	−0.213	0.053	15.904	<0.001	0.808	0.728	0.898	0.928	1.078

CRP, Canalith Repositioning Procedure; 25-OH-VD, 25-hydroxyvitamin D.

### 3.2 Comparison of serum calcium with 25-OH-VD

25-OH-VD and calcium levels in the control group were significantly higher than those in the BPPV non-recurrence group and the BPPV recurrence group, with statistically significant differences (*p* < 0.05). 25-OH-VD and serum calcium levels in the BPPV non-recurrence group were significantly greater than those in the BPPV recurrence group (*p* < 0.05) (Table 3).

**Table 3:** Comparison of serum calcium with 25-OH-VD.

Group	*N*	serum calcium(mmol/L)	25-OH-VD (ng/ml)
Control group	60	2.68 ± 0.48	23.61 ± 3.92
BPPV No recurrence group	213	2.35 ± 0.56^*^	18.44 ± 3.74^*^
BPPV Relapse group	73	1.94 ± 0.47^*#^	15.34 ± 3.45^*#^
*F*		33.145	82.910
*p*		<0.001	<0.001

BPPV, benign paroxysmal positional vertigo; 25-OH-VD, 25-hydroxyvitamin D. *p < 0.05 vs control group; #*p* < 0.05 vs BPPV non-recurrence group.

Post-hoc analysis revealed significant differences in 25-OH-VD levels between all pairwise comparisons: control vs. non-recurrence group (*p* < 0.001), control vs. recurrence group (*p* < 0.001), and non-recurrence vs. recurrence group (*p* < 0.001), (Figure 2).

**Figure 2:**
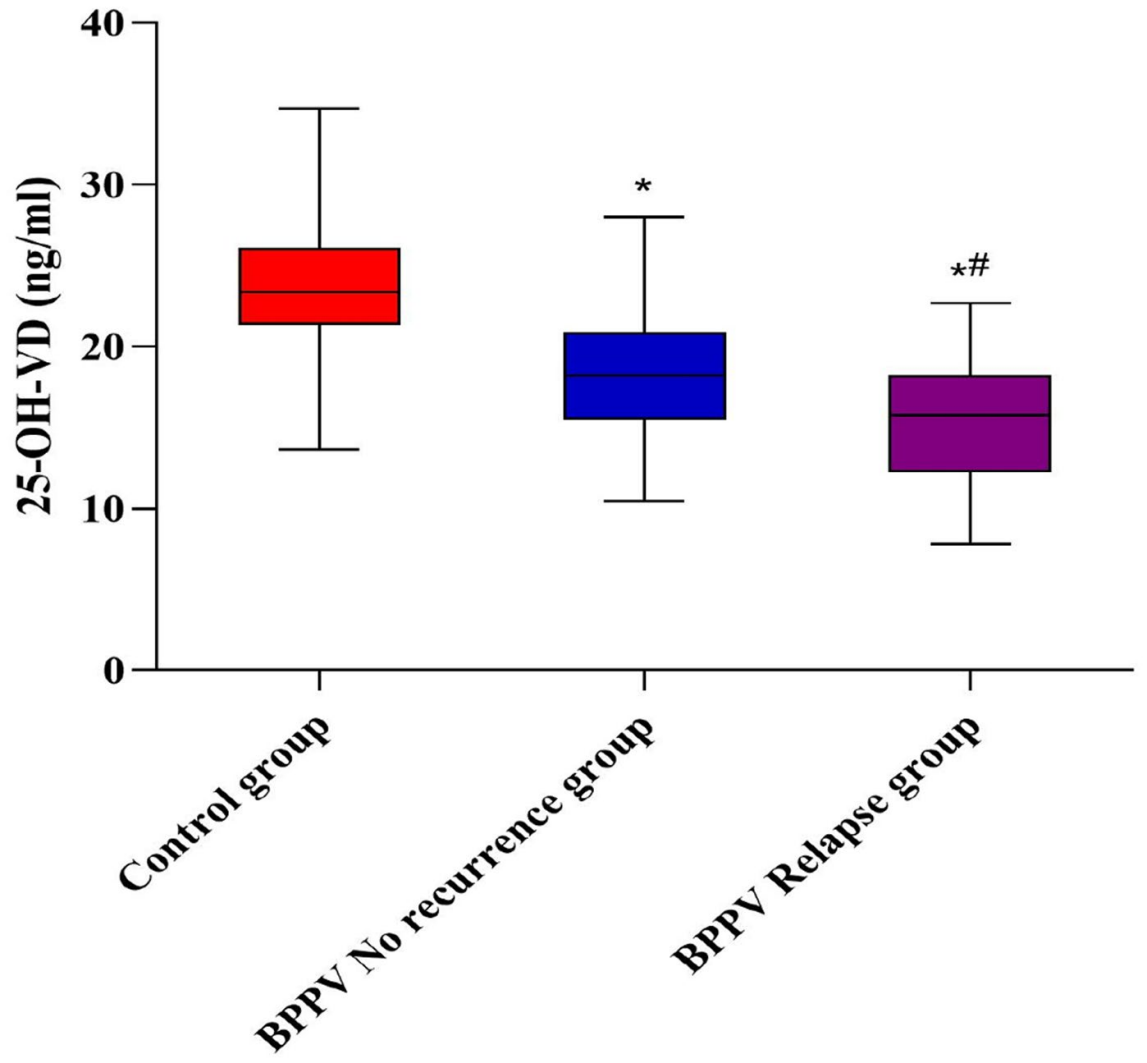
Boxplot of 25-OH-VD levels in control, BPPV non-recurrence, and BPPV recurrence groups. *p < 0.05 vs. control group; #*p* < 0.05 vs. BPPV non-recurrence group.

### 3.3 Correlation analysis of 25-OH-VD and serum calcium in elderly patients with recurrent BPPV

In elderly patients with recurrent BPPV (73 cases), serum calcium was significantly *p*ositively correlated with 25-OH-VD (*r* = 0.637, *p* < 0.05, Figure 3).

**Figure 3:**
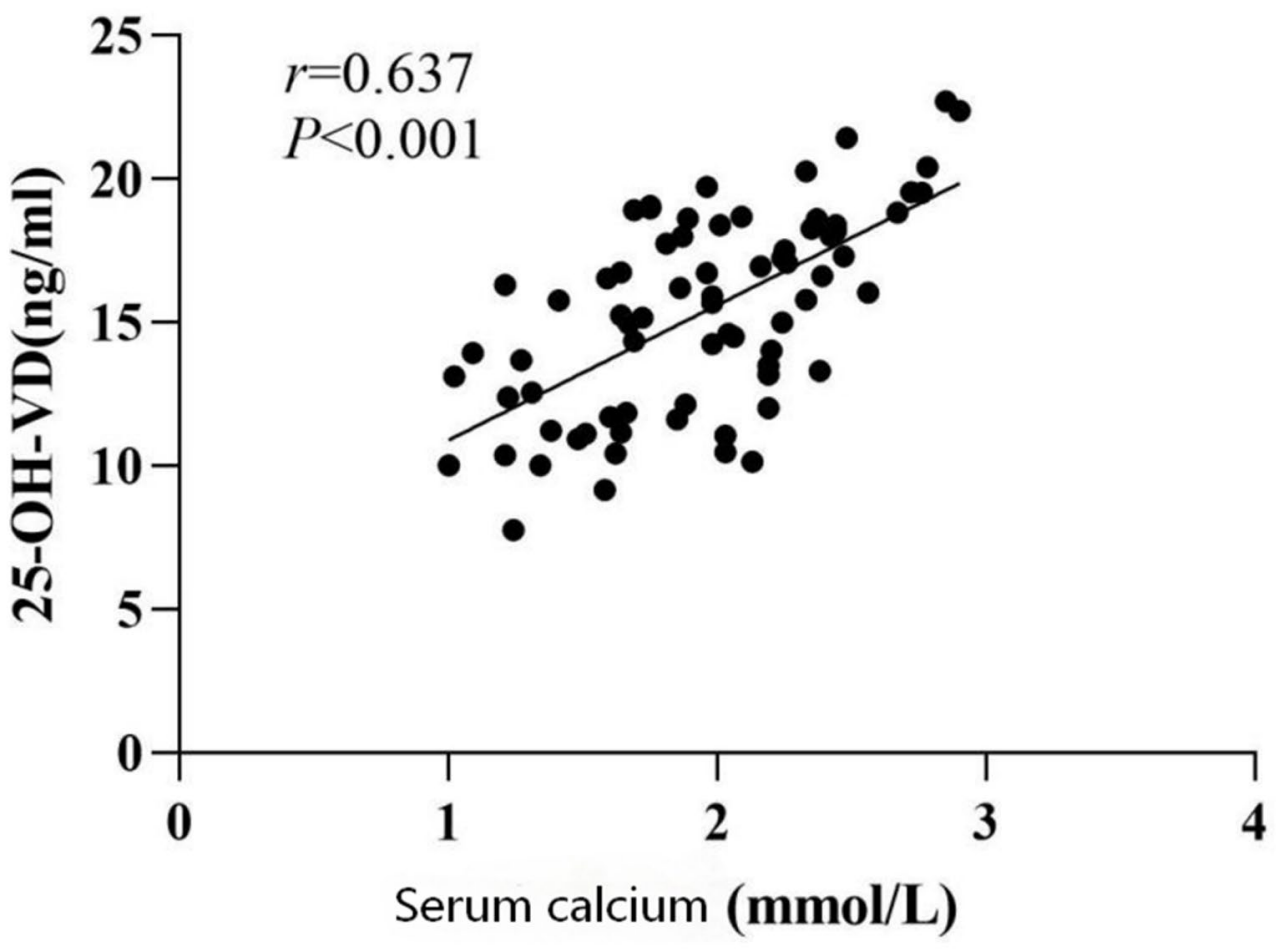
Correlation analysis of serum calcium concentration and 25-OH-VD in elderly patients with recurrent BPPV. BPPV, Benign Paroxysmal Positional Vertigo; 25-OH-VD, 25-Hydroxyvitamin D.

### 3.4 ROC curve analysis to assess the predictive value of serum calcium concentration and 25-OH-VD levels for recurrence of BPPV in elderly patients

Serum calcium and 25-OH-VD are standardized biochemical markers that provide objective predictive thresholds for clinical use. These two indicators were included in logistic regression analysis to construct a predictive model for recurrence in elderly BPPV patients. The individual prognostic index (PI) equation is: PI = −1.149X₁– 0.216X₂ + 5.046, where X₁is serum calcium and X₂is 25-OH-VD. PI represents the logit value from a bivariate regression model combining only these two biomarkers; thus, the coefficients differ from those in the multivariate model (Table 2) and are intended solely for their combined calculation. A lower PI value indicates a higher recurrence risk. Based on the maximum Youden index (0.374), the optimal cutoff for PI was 0.339, indicating a higher probability of recurrence when PI < 0.339 (Figure 4; Table 4). The optimal cutoff values for single indicators were 2.45 mmol/L for serum calcium and 18.98 ng/mL for 25-OH-VD, with the combined model showing superior predictive value (AUC = 0.762 vs. 0.707 for serum calcium and 0.717 for 25-OH-VD). The serum calcium cutoff should be interpreted in conjunction with the laboratory reference range (2.11–2.52 mmol/L). Internal validation using 1,000 Bootstrap resamples demonstrated good model performance: calibration intercept = 0.018, calibration slope = 0.986; Brier score = 0.1185; decision curve analysis showed net benefit at 3–97% threshold probabilities.

**Figure 4:**
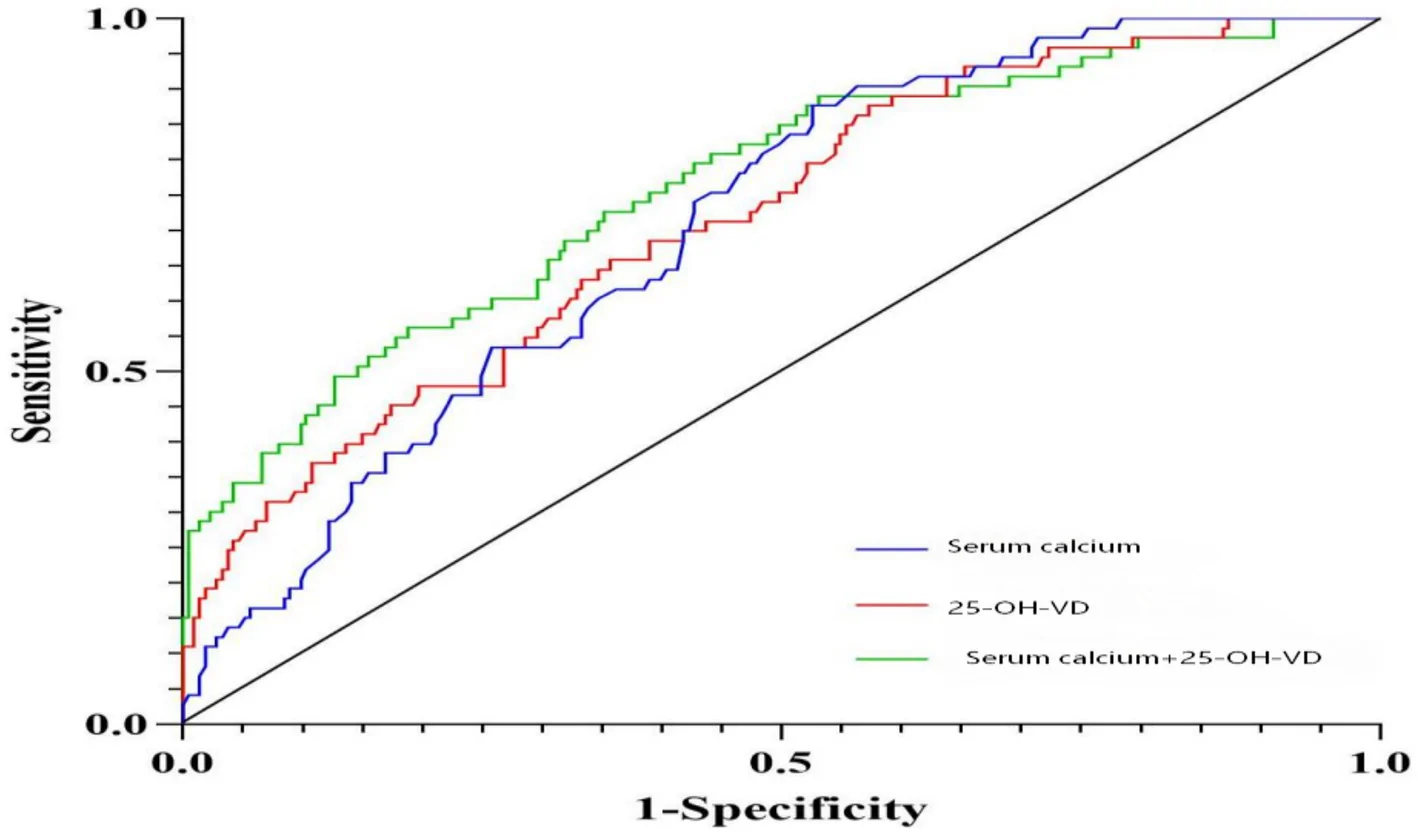
ROC curve evaluating the predictive value of serum calcium and 25-OH-VD for recurrence in elderly BPPV. BPPV, Benign Paroxysmal Positional Vertigo; 25-OH-VD, 25-Hydroxyvitamin D; ROC, Receiver Operating Characteristic; AUC, Area Under the Curve; CI, Confidence Interval.

**Table 4:** ROC curve assessment of the predictive value of 25-OH-VD and serum calcium for recurrence in BPPV elderly patients.

Variables	AUC	95%CI	*p*	Sensitivity (%)	Specificity (%)	Youden index	Cutoff value
Serum calcium	0.707	0.644–0.769	<0.001	87.7	47.4	0.351	2.45
25-OH-VD	0.717	0.651–0.783	<0.001	87.7	42.7	0.304	18.98
Serum calcium +25-OH-VD	0.762	0.697–0.827	<0.001	56.2	81.2	0.374	0.339

Internal validation results: calibration intercept = 0.018, calibration slope = 0.986; Brier score = 0.1185; decision curve analysis showed net benefit at 3–97% threshold probabilities (Figure 5).

**Figure 5:**
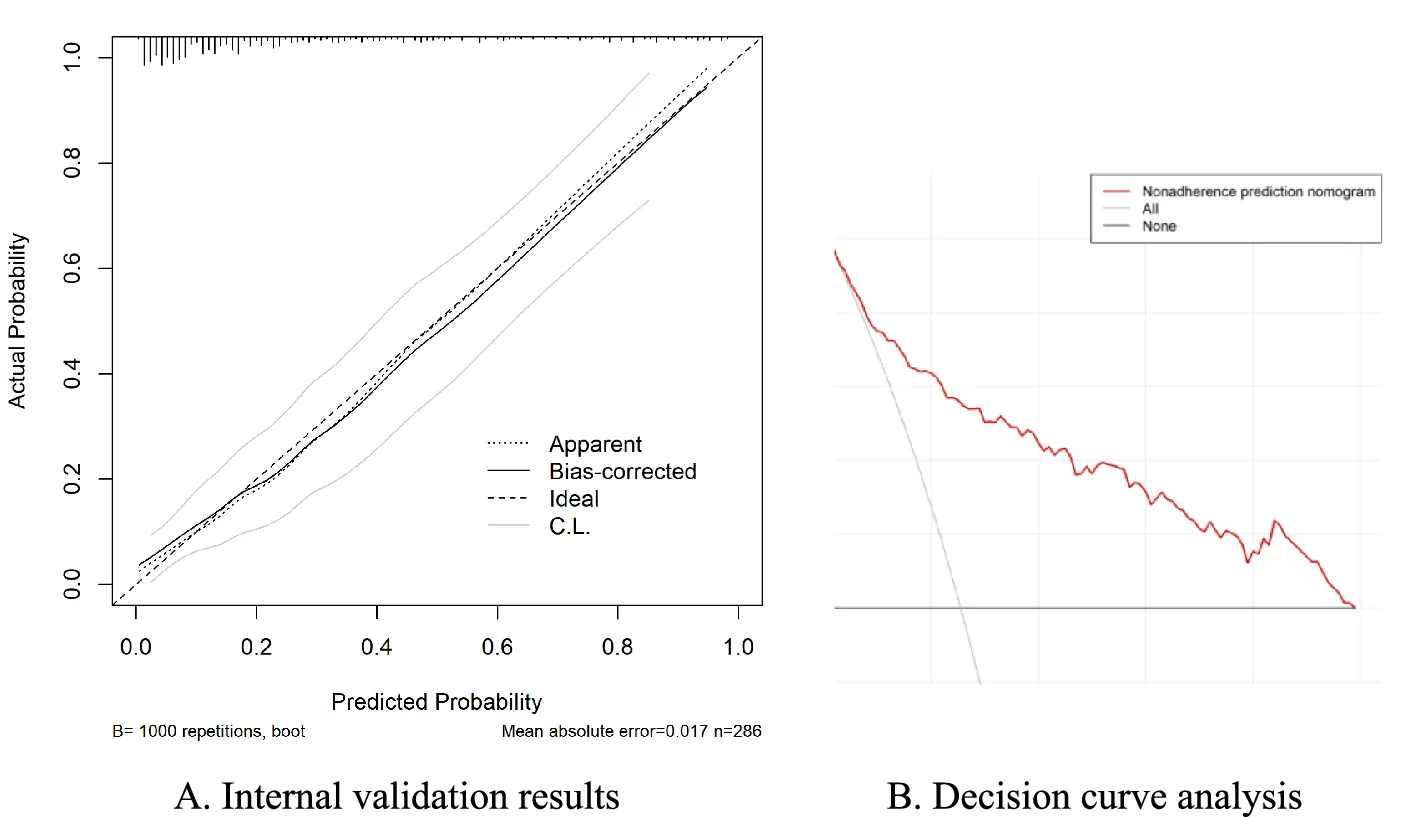
Internal validation of the combined predictive model for recurrence of BPPV in elderly patients. **(A)** Internal validation results. **(B)** Decision curve analysis (DCA).

## 4 Discussion

This study, using a single-center follow-up cohort study design, systematically analyzed the predictive value of 25-OH-VD and serum calcium for recurrence after CRP in elderly patients with BPPV. The results showed that elderly BPPV patients often had multiple repositioning attempts, hypertension, hyperglycemia, hyperlipidemia, sleep disorders, cervical spondylosis, anxiety, and decreased 25-OH-VD and serum calcium levels. More importantly, this study confirmed that 25-OH-VD and serum calcium are independent predictors of recurrence after CRP, and their combined predictive power is even higher. These findings not only provide important biomarkers for early clinical identification of patients at high risk of recurrence but also offer new insights into the prevention and treatment of recurrence, particularly in the context of an aging society, and have potential value in reducing the burden on healthcare.

### 4.1 Clinical characteristics and comorbidity burden of elderly patients with BPPV

This research concluded that the prevalence of hypertension, hyperlipidemia, and hyperglycemia in BPPV patients was significantly higher than in the control group and this finding is in line with several earlier studies (33, 34). This metabolic syndrome clustering effect indicates that BPPV is not merely a mechanical dislocation of otoconia, but a local expression of the systemic metabolic condition in the inner ear. Moreover, the possible mechanisms by which elderly BPPV patients are often accompanied by multiple metabolic abnormalities (35–37): (1) Long-term hypertension may cause inner ear microvascular lesions, leading to insufficient blood supply to vestibular organs and affecting otoconia stability; (2) metabolic disorders in diabetic patients may cause changes in the pH value of endolymph, accelerating the dissolution of calcium carbonate crystals in otoconia; (3) abnormal lipid metabolism may affect hemorheological properties, leading to inner ear microcirculation disorders. In addition, this study also observed that the proportions of sleep disorders, cervical spondylosis, and anxiety in the experimental group were significantly increased. Both univariate and multivariate regression analyses confirmed these factors as independent influencing factors for recurrence. Sleep disorders can either worsen the vestibular compensation disorders by autonomic nervous dysfunction, or can interact with anxiety to create a vicious cycle (38). Cervical spondylosis is a prevalent condition in the geriatric population that can limit the movement of the neck due to degenerative alterations of the cervical spine or directly squeeze the vertebral artery, disrupting inner ear microcirculation and thus worsening otoconia instability (39). The fear of vertigo attacks is often the source of anxiety, which causes limited activity and a greater psychological load, slowing down the recovery process (39). These comorbidities indicate that the recurrence process of BPPV is multi-system, and clinical treatment should not be limited to a single repositioning procedure but should transition to a multi-system approach that incorporates sleep, skeletal, and psychological health evaluation.

### 4.2 Abnormalities in calcium and vitamin D metabolism and their predictive efficacy

It is noteworthy that the core outcome of this research is that patients in the experimental group have significant calcium metabolism abnormalities. The serum calcium and 25-OH-VD levels are both substantially lower than those of the control group, and most patients are in a state of vitamin D insufficiency (<20 ng/mL). This outcome is in line with the findings of the study by Ren et al., whose research showed that vitamin D deficiency is commonly present in BPPV patients (40). In the elderly population, due to age-related changes, reduced outdoor activity, decreased skin synthesis capacity, and insufficient dietary intake, the incidence of vitamin D deficiency is relatively high, which may partially explain the high occurrence of BPPV in the elderly population (14).

ROC curve analysis further confirmed the excellent value of these two indicators in clinical prediction. The AUC of serum calcium was 0.707 and the optimal cutoff value was 2.45 mmol/L; the AUC of 25-OH-VD was 0.717 and the optimal cutoff value was 18.98 ng/mL. More to the point, the AUC of the combined diagnosis of the two raised to 0.762, and the specificity was as high as 81.2. This implies that combined testing is more effective in identifying the really high-risk groups and minimizing misdiagnosis. It is interesting to note that the best cutoff value of 25-OH-VD in this study (18.98 ng/mL) is quite near the clinically determined cutoff of vitamin D deficiency (<20 ng/mL). This small variation has significant clinical warning implications: even in the range of the so-called borderline insufficiency that is typically taken into account, the risk of recurrence of elderly BPPV patients has already started to grow dramatically. This gives a more specific intervention target to clinical practice, indicating that in BPPV patients, vitamin D levels in the adequate range (>20 ng/mL) may be a precondition to preventing recurrence.

### 4.3 Pathophysiological mechanisms

Vitamin D is implicated in the inner ear calcium homeostasis regulation in several ways. To begin with, the vitamin D receptor (VDR) and calcium-binding proteins are extensively expressed in the vestibular dark and stria vascularis cells of the inner ear. Vitamin D controls the expression of epithelial calcium channels (including ECaC1) through VDR activation, thus directly influencing the level of endolymphatic calcium (10). This study revealed that the level of 25-OH-VD in the recurrence group was significantly lower than in the non-recurrence group, which is strong clinical data to support the hypothesis that vitamin D plays a role in the occurrence of recurrence. Otoconia are primarily made of glycoprotein matrix and calcium carbonate crystals and their stability is extremely reliant on local calcium ion concentration and metabolic balance. Deficiency in vitamin D can cause mineral composition abnormalities in otoconia, which are reflected in uneven crystal size, abnormal morphology, and even micro-dissolution (9). These abnormal otoconia particles may persist after CRP or be more prone to re-detachment due to weakened attachment, potentially leading to recurrence.

In addition, the occurrence of recurrence is not only related to pathological changes of the peripheral vestibular organs, but also involves the functional state of the central vestibular system. Vitamin D, as a neuroactive steroid, may regulate the central vestibular compensation process by affecting the expression of neurotrophic factors and synaptic plasticity (40, 41). Animal experiments have shown that vestibular compensation ability is significantly impaired in vitamin D-deficient rats, which suggests that vitamin D deficiency may delay the remodeling of vestibular function, causing Recurrence symptoms to persist or recur. Therefore, maintaining adequate vitamin D levels is equally crucial for promoting functional recovery of the central nervous system.

It should be noted that 25-OH-VD and serum calcium are biologically correlated, as vitamin D regulates calcium absorption and metabolism. While this correlation was accounted for in the multivariate model, the combined predictive index should be interpreted with caution in clinical practice.

### 4.4 Comparison with previous studies and innovative points

The findings are both in line with previous studies and also have differences in extension. Consistent with the study of Chua et al. (14), this study also found a strong correlation between vitamin D and BPPV, but this study further extends the research focus from “incidence” to “recurrence prediction,” which is an important extension of the previous research field. Relative to the previous studies that only focused on a single indicator (42), this study not only confirmed the relation between BPPV and vitamin D, but also determined the optimal cutoff value for predicting recurrence and a combined prediction model through multivariate regression and ROC curve analysis. This quantitative risk assessment tool provides a practical approach for risk stratification in primary care settings, which may assist clinicians in identifying high-risk patients who require closer follow-up.

### 4.5 Limitation

The limitations of this study are as follows: (1) The single-center design may limit the generalizability of the findings, and future multi-center, large-sample studies are needed to validate these results. (2) Serum calcium and 25-OH-VD levels were measured only at baseline without dynamic follow-up, precluding assessment of their changes during recurrence. (3) Additional potential predictors of recurrence, including vestibular function parameters and bone mineral density, were not evaluated. Prospective interventional studies incorporating vestibular assessments and imaging are needed to further explore recurrence mechanisms. (4) The combined predictive model had relatively low sensitivity (56.2%), meaning that nearly half of recurrent cases would be missed if used alone. Therefore, it should be interpreted as an adjunctive tool rather than a standalone diagnostic test. (5) The single-center follow-up cohort design cannot establish causality. The observed association between low serum calcium, low 25-OH-VD, and BPPV recurrence may reflect reverse causation, confounding, or both. For instance, patients with recurrent BPPV may have reduced outdoor activity or dietary changes that lower vitamin D levels. Randomized controlled trials are needed to determine whether vitamin D and calcium supplementation can truly reduce the risk of BPPV recurrence. (6) Although patients with major comorbidities affecting calcium and vitamin D metabolism were excluded, several potential confounding factors were not systematically measured or adjusted for, including seasonal variation, sunlight exposure, dietary calcium/vitamin D intake, serum albumin levels, and detailed parathyroid function. These unmeasured confounders may influence serum calcium and 25-OH-VD levels, and their impact on BPPV recurrence cannot be ruled out. Therefore, the observed associations should be interpreted with caution, and no strong causal or interventional conclusions can be drawn from this observational study. Randomized controlled trials are warranted to confirm whether vitamin D and calcium supplementation can truly reduce recurrence risk.

## 5 Conclusion

Serum calcium and reduced 25-OH-VD are independent predictive factors for post-CRP recurrence in elderly BPPV patients, and their combination improves predictive efficacy. The predictive model provides a quantitative basis for early identification of high-risk patients. However, due to its relatively low sensitivity, the model should be used as an adjunctive tool rather than a standalone test in clinical practice. These findings require further external validation in multi-center studies, and interventional effects of calcium and vitamin D supplementation should be confirmed by randomized controlled trials.

## Data Availability

The original contributions presented in the study are included in the article/supplementary material, further inquiries can be directed to the corresponding author.
